# Composition-on-composition regression analysis for multi-omics integration of metagenomic data

**DOI:** 10.1093/bioinformatics/btaf387

**Published:** 2025-07-12

**Authors:** Nicholas Rios, Yuke Shi, Jun Chen, Xiang Zhan, Lingzhou Xue, Qizhai Li

**Affiliations:** Department of Statistics, George Mason University, Fairfax, VA 22030, United States; State Key Laboratory of Mathematical Sciences, Academy of Mathematics and Systems Science, Chinese Academy of Sciences, Beijing 100190, China; Biomedical Statistics and Informatics, Mayo Clinic, Rochester, MN 55905, United States; School of Statistics and Data Science, Southeast University, Nanjing 211189, China; Department of Statistics, Pennsylvania State University, University Park, PA 16802, United States; State Key Laboratory of Mathematical Sciences, Academy of Mathematics and Systems Science, Chinese Academy of Sciences, Beijing 100190, China; School of Mathematical Sciences, University of Chinese Academy of Sciences, Beijing 101408, China

## Abstract

**Motivation:**

Compositional data are frequently encountered in many disciplines, such as in next-generation sequencing experiments widely used in biomedical studies. Regression analysis with compositional data as either responses or predictors has been well studied. However, when both responses and predictors are compositional, the inventory of analysis tools is surprisingly limited, especially in the high-dimensional setting. Among the few existing methods, most of them rely on a log-ratio transformation to move compositional data from the simplex to real numbers. Yet, a serious weakness of these methods is their failure to handle the substantial fraction of zeroes observed in data collected from next-generation sequencing experiments.

**Results:**

To investigate associations between two high-dimensional multi-omics compositions, we propose a composition-on-composition (COC) regression analysis method which does not require log-ratio transformations and hence can handle zeroes in the data. To account for high dimensionality, we estimate regression coefficients using a penalized estimation equation approach. Finally, inference procedures for COC regression are also proposed. Superior performance of COC is demonstrated through both comprehensive numerical simulations and case studies.

**Availability and implementation:**

Source R codes to implement COC method is available at https://github.com/nrios4/COC.

## 1 Introduction

Next-generation sequencing (NGS) data, employed extensively in various domains of modern biological and biomedical research (e.g. 16S rRNA microbiome surveys, single-cell RNA-seq studies, and other omics studies), are inherently compositional, providing only relative abundance information (Gloor *et al.* 2016, 2017, Vandeputte *et al.* 2017). In the realm of biomedical research, it is frequently observed that the aforementioned compositional data are collected from multiple sources pertaining to the same subjects. This may include microbiome compositional data from various body sites or RNA-seq data from different tissue samples belonging to the same individuals. Additionally, it is common for researchers to gather more than one type of NGS “omics” data—such as microbiome and metabolomics data—to explore their interactions and combined effects on human disease conditions (Morton *et al.* 2019a). In both scenarios, it is interesting to explore whether dependencies exist among these disparate NGS compositional datasets. Integrating these multi-omics compositional datasets is crucial to understand complex biological processes and disease mechanisms at a deeper level. Unfortunately, most existing methods for combining multi-omics data focus on a single pair of two omics features each time and then aggregate these marginal results to obtain a final conclusion (Morton *et al.* 2019a). However, ignoring the compositional nature of these multi-omics data may lead to suboptimal or sometimes misleading results, including elevated false discovery rates and low reproducibility rates (Gloor *et al.* 2017, Hawinkel *et al.* 2019, Morton *et al.* 2019b, Nearing *et al.* 2022). To address these limitations, we are investigating an integrative multi-omics analysis approach to infer interactions across two compositional omics datasets in this article.

In traditional regression analysis involving compositional predictors, log-ratio transformations are commonly employed (Aitchison 1982). These transformed log-ratios, no longer compositional, have found extensive application in subsequent statistical modeling and analysis. For instance, they are integral to the linear log contrast model utilized in regression analysis with a univariate outcome and high-dimensional compositional predictors (Aitchison and Bacon-Shone 1984, Lin *et al.* 2014, Shi *et al.* 2016, Li *et al.* 2023). Methods based on these transformations have been further developed for multivariate regression models with compositional outcomes (Chen *et al.* 2017, Alenazi 2021, Fiksel *et al.* 2022). However, these methods are not fully suited for high-dimensional NGS compositional data by falling short in accommodating compositional data containing zeros, which are common occurrences in NGS datasets (Jiang *et al.* 2023, Chan and Li 2024). The only exception is the direct regression (DR) method proposed in Fiksel *et al.* (2022), which is transformation-free and hence can handle zero values. Unfortunately, the DR method was proposed under low-dimensional settings and its performance with high-dimensional compositional predictors/responses is largely unknown. In response to this gap, we propose a novel high-dimensional composition-on-composition (COC) regression analysis that is designed to equip practitioners with valid statistical analysis and inference tools for applications involving such complex NGS data structures (i.e. compositional, zero-inflated, high-dimensional). The novelty of COC resides in being the inaugural method of its kind that not only exhibits advantageous properties in model estimation and prediction but also addresses a critical application gap for which existing statistical analysis methods have been unsatisfactory.

Inspired by Fiksel *et al.* (2022), we adopt the same DR framework with both responses and predictors being compositional in order to handle zero values in NGS data in our COC approach. Beyond the issue of excessive zeros, another significant computational hurdle is the estimation of a vast number of regression coefficients, a task complicated by the extensive array of omics features typically uncovered in both microbiome studies and many other NGS experiments. To enhance model estimation in such contexts, we introduce a novel penalty function that is not only biologically insightful but also statistically robust, leading to more precise model estimates and predictions. We have developed an efficient algorithm for solving the model’s penalized optimization problem. Furthermore, we explore a statistical inference procedure to assess the uncertainties related to COC model estimates and predictions. Our simulation studies indicate that the COC approach surpasses existing methods in terms of model estimation and inference accuracy. Moreover, we apply our COC analysis method to examine the interactions between bacterial and fungal communities within the gut microbiota, utilizing compositional data from the COMBO study (Wu *et al.* 2011). Our findings reveal associations between three bacterial genera (*Prevotella, Bacteroides, Lachnospiraceae I.S.*) and fungal compositions, aligning partially with prior research that highlighted the significance of the *Prevotella/Bacteroides* ratio in substantial correlation with fungal compositions.

The remainder of this article is organized as follows. In Section 2, we first review the COC regression framework and then propose a novel penalized estimation equation approach to fit COC model in high-dimensional settings. Inference procedures on COC predictions are also discussed. We present comprehensive simulation studies across different scenarios to validate empirical properties of COC and apply it to analyze real datasets collected from motivating studies to draw biological insights in Section 3. This article concludes with a brief discussion in Section 4.

## 2 Materials and methods

### 2.1 The COC regression model

Let **x** and **y** be a *p*-dimensional and a *q*-dimensional compositional vectors from simplex $S^{p}=\{{\left(x_{1},\ldots ,x_{p}\right)}^{T}:\sum_{j=1}^{p}x_{j}=1,x_{j}\geq 0\}$ and $S^{q}=\{{\left(y_{1},\ldots ,y_{q}\right)}^{T}:\sum_{k=1}^{q}y_{k}=1,y_{k}\geq 0\}$, respectively, where *p* and *q* can be potentially larger than the sample size. That is, we are particularly interested in high-dimensional settings to mimic the environment of high-throughput NGS compositional data. To enhance model interpretability and to handle zeros, a DR framework has been proposed to model relations between **x** and **y** (Fiksel *et al.* 2022) using the following model:

(1) E(y|x)=Bx,

where $B=\{B_{kj}\}$ is a q×p matrix of regression coefficients. To guarantee that the expected outcomes under model (1) are compositional, it must be true that $1=\sum_{k=1}^{q}E(y_{k}|x)=\sum_{k=1}^{q}\sum_{j=1}^{p}B_{kj}x_{j}$ holds for all compositional predictors **x**. An equivalent condition would be that $\sum_{k=1}^{q}B_{kj}=1$ must hold for each j=1,…,p. To guarantee that the expected outcomes are nonnegative for all compositional predictors, we also need all entries in **B** to be nonnegative. Therefore, matrix **B** needs to satisfy the following conditions in order to make model (1) well-defined:

(2) $$B\in \{R^{q\times p}:B_{kj}\geq 0,\sum_{k=1}^{q}B_{kj}=1,\;\;\;\;\forall \;\;\;\;j=1,\ldots ,p\}.$$

An intuitive understanding of condition (2) can be obtained by treating a compositional vector as the probability mass function (PMF) of a discrete random variable. Then, **B** transfers PMF **x** to PMF E(y|x) in Equation (1). That is, **B** is a rectangular Markov matrix and reduces to a Markov matrix if p=q. Unlike log-ratio transformations, zero coordinates in either predictors **x** or responses **y** are allowed under model (1).

In ordinary multivariate linear regression, one can view regression coefficient $B_{kj}$ as the effect of predictor $x_{j}$ on response $y_{k}$. However, in COC regression, such a regression coefficient interpretation is less meaningful since each component of a compositional vector only carries relative information about the whole composition. A more appropriate interpretation of these regression coefficients is as follows. Let $\delta \leq \min(1-x_{j},x_{j^{\prime }})$, and suppose $x_{j}$ increases by δ units at the expense of $x_{j^{\prime }}$ decreasing by δ units, while holding the rest of *x*-components constant. Then, the conditional expectations E(y|x) change by $\delta (B_{\cdot j}-B_{\cdot j^{\prime }})$, where $B_{\cdot j}$ denotes the *j*th column of **B**.

### 2.2 Model estimation

To estimate **B** in COC regression, we employ the estimation equation method and seek an objective Q(B) that fulfills the condition E(▽BQ(B))=0. In particular, a suitable function meeting this criterion is the Kullback–Leibler divergence (KLD) between the compositional vectors **y** and E(y|x) (Fiksel *et al.* 2022), which is defined as:

$$\mathrm{KLD}(y,E(y|x))=-\sum_{i=1}^{n}\sum_{k=1}^{q}y_{ik}\;\text{log}\;\left(\frac{\sum_{j=1}^{p}x_{ij}B_{kj}}{y_{ik}}\right).$$

The objective is to minimize the KLD under the constraint that B∈F, where $F=\{B\in R^{q\times p}:B_{kj}\geq 0,\sum_{k=1}^{q}B_{kj}=1,\forall \;\;\;\;j=1,\ldots ,p\}$. An EM algorithm has been developed to solve this problem, facilitating the derivation of versatile estimators under the low-dimensional scenario (Fiksel *et al.* 2022). However, it may not be suitable for high-dimensional settings often associated with high-throughput NGS data considered in this study. To estimate a substantial number of parameters in the regression coefficient matrix **B**, sparsity assumptions are necessary for stable and precise estimation. In high-dimensional statistics, one common way to obtain a reasonable estimator is to shrink small coefficients to zero using sparsity-promoting penalties (e.g. Lasso). Unfortunately, under the COC framework, this operation probably leads to a solution $\hat{B}$ that does not belong to the feasible set F. Therefore, given the unique structure of **B** in Equation (2), it is imperative to carefully design a penalty function that not only encourages sparsity in **B** but also has interpretability within the COC regression framework.

In a traditional linear regression model y=Xβ+ϵ, the coefficient $\beta_{j}$ measures the effect of *j*th predictor $x_{j}$ on the response, and a typical sparsity assumption in a high-dimensional linear model requires that $\beta_{j}=0$ for most *j*’s. However, in our COC regression model, the coefficients $B_{.j}$ have no direct interpretations since compositional predictors are all relative. On the other hand, the joint effect of $\left(x_{j},x_{j^{\prime }}\right)$ (in the sense that $x_{j}$ increases by δ units at the expense of $x_{j^{\prime }}$ decreasing by δ units, while holding the remaining coordinates constant) on **y** are characterized by $\delta (B_{\cdot j}-B_{\cdot j^{\prime }})$. That is, $B_{.j}-B_{.j^{\prime }}$ can measure the relative effect of predictor pair ($x_{j},x_{j^{\prime }}$). Analogous to sparsity assumptions in linear regression, it would make sense that for most pairs $\left(j,j^{\prime }\right)$, sparsity implies that $B_{\cdot j}-B_{\cdot j^{\prime }}=0$ for COC regression. Given that each column of **B** belongs to the simplex $S^{q}$, where the null element in $S^{q}$ is represented by a vector of all 1/q’s in the Aitchison geometry (Aitchison 1982), it is logical to consider shrinking the majority of **B**’s columns towards this null element ${\left(\frac{1}{q},\ldots ,\frac{1}{q}\right)}^{T}$. This can be accomplished by applying column-wise sparsity penalties. Consequently, we define the column-wise centered matrix $\tilde{B}$ as ${\tilde{B}}_{kj}=B_{kj}-\frac{1}{q}$ for k=1,…,q and j=1,…,p, and employ the $L_{2,1}$-norm of $\tilde{B}$ as the new penalty term used in COC regression:

$$||\tilde{B}|{|}_{2,1}=\sum_{j=1}^{p}||{\tilde{B}}_{.j}|{|}_{2}=\sum_{j=1}^{p}\sqrt{\sum_{k=1}^{q}{\tilde{B}}_{kj}^{2}},$$

where $\tilde{B}.j$ denotes the $j^{th}$ column of $\tilde{B}$.

Integrating this penalty function with the previous KLD objective, we introduce a novel strategy to estimate the regression coefficients matrix **B** by solving the following penalized objective:

(3) $$\hat{B}=\underset{B\in F}{\text{argmin}}-\sum_{i=1}^{n}\sum_{k=1}^{q}y_{ik}\;\text{log}(\sum_{j=1}^{p}x_{ij}B_{kj})+\lambda \sum_{j=1}^{p}\sqrt{\sum_{k=1}^{q}{\tilde{B}}_{kj}^{2}},$$

where λ≥0 acts as a tuning parameter, balancing the trade-off between goodness-of-fit and model complexity. A computing algorithm to solve Equation (3) along with further discussion on COC are presented in Section A of the online supplementary data at *Bioinformatics* online. As the first attempt in the high-dimensional COC regression framework, the penalty function used in Equation (3) is one possible solution but might not be optimal under certain criteria. However, as will be seen later in this article, numerical studies will demonstrate that this penalty function does significantly improve model estimation and prediction performance under a wide range of scenarios in high-dimensional settings (even when the sparsity assumption implied in our penalty function is violated) compared to a corresponding approach (Fiksel *et al.* 2022) without any penalization.

### 2.3 Model inference

The next step is to quantify uncertainties about the estimator and to perform statistical inference. Since COC does not specify an exact distribution for compositional responses, estimating uncertainties associated with $\hat{B}$ can be done by using resampling strategies (e.g. bootstrap or permutation tests). Considering the interpretation of **B** in COC regression, it is of interest to determine whether the columns of **B** are equal. Let $B_{0}$ be a q×p matrix whose entries are all 1/q. Then, we can test $H_{0}:B=B_{0}$. Fiksel *et al.* (2022) proposed a permutation test for this type of null hypothesis and can be applied to this high-dimensional setting. Alternatively, we propose a bootstrap-based hypothesis test of $H_{0}$ which is a modified version of a similar test in Rios *et al.* (2024). This algorithm uses the inverse perturbation operator in the Aitchison geometry (Pawlowsky-Glahn and Egozcue 2006) to simulate the distribution of the compositional response under $H_{0}$. This operator is defined as $a⊖b=(a_{1}/b_{1},\ldots ,a_{p}/b_{p})/(\sum_{i=1}^{p}a_{i}/b_{i})$ for compositional vectors $a,b\in S^{p}$ and all **b**-components being positive. The proposed test is summarized in Algorithm 1.

**Algorithm 1:** Bootstrap Hypothesis Test for $H_{0}:B=B_{0}$.

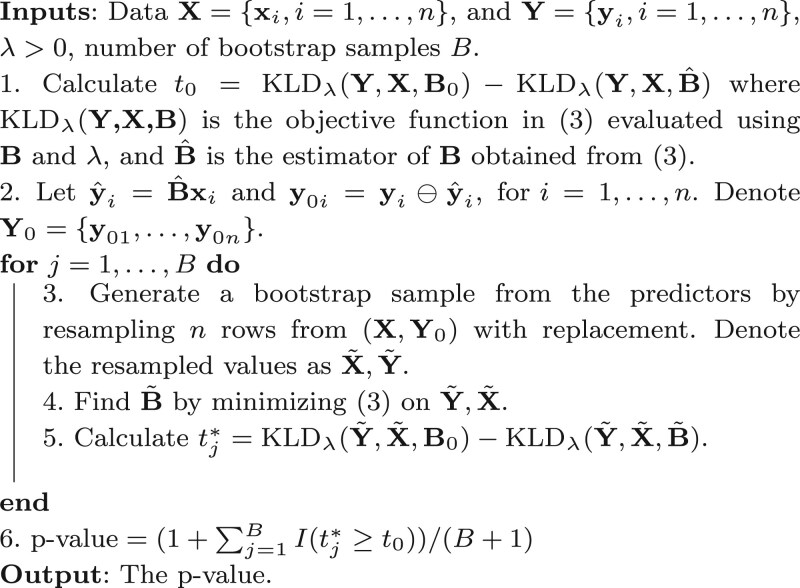

It is also of interest to quantify the uncertainty of model predictions. Specifically, it would be ideal to have a confidence region for the compositional responses given a new predictor $x_{n+1}$, which has not been investigated previously (Fiksel *et al.* 2022). In particular, we use the conformal inference framework (Shafer and Vovk 2008, Lei *et al.* 2018) to provide prediction regions for new observations. Suppose we have a new predictor observation $x_{n+1}$ and a candidate value $y_{\mathrm{cand}}\in S^{q}$. The main idea is that we can measure the “conformity” of $y_{\mathrm{cand}}$ to existing data via the KLD. Construct an augmented dataset $\left(x_{1},y_{1}),\ldots ,(x_{n},y_{n}),(x_{n+1},y_{n+1}\right)$, where $y_{n+1}=y_{\mathrm{cand}}$ for ease of notation. Let ${\hat{y}}_{i}$ be the predicted composition for $x_{i}$ under the proposed COC method, fitted to the augmented dataset $\left(x_{i},y_{i}\right)$ for i=1,…,n+1. Then, one can define:

(4) $$\pi (y_{\mathrm{cand}})=\frac{1}{n+1}\sum_{i=1}^{n+1}I(R_{i}\leq R_{n+1}),$$

where $R_{i}=\text{KLD}(y_{i},{\hat{y}}_{i}),i=1,\ldots ,n+1$ are the conformity scores. Under the assumption of exchangeability for data $(x_{i},y_{i}),i=1,\ldots ,n+1$, it can be shown that $\left(n+1)\pi (y_{\mathrm{cand}}\right)$ is uniformly distributed over 1,2,…,n+1 (Lei *et al.* 2018). Hence

$$P\left((n+1)\pi (y_{\mathrm{cand}})\leq \left\lceil \left(1-\alpha )(n+1\right)\right\rceil \right)\geq 1-\alpha ,$$

where ⌈x⌉ maps *x* to the least integer greater than or equal to *x*. A 100(1−α)% prediction region for $y_{n+1}$ can then be defined as:

(5) $$C_{1-\alpha }=\{y_{\mathrm{cand}}\in S^{q}:(n+1)\pi (y_{\mathrm{cand}})\leq \left\lceil \left(1-\alpha )(n+1\right)\right\rceil \}.$$

Assuming samples $(x_{i},y_{i}),i=1,\ldots ,n$ are independent and identically distributed, then it has been shown that $P(y_{n+1}\in C_{1-\alpha })\geq 1-\alpha$ for any new independent and identically distributed pair $\left(x_{n+1},y_{n+1}\right)$ (Vovk *et al.* 2005, Lei *et al.* 2018). To construct a prediction region, one would need to repeat the steps in Equations (4) and (5) for many different candidates $y_{\mathrm{cand}}\in S^{q}$ to visualize $C_{1-\alpha }$, which is computationally infeasible if the dimension *q* is large. Fortunately, a computationally efficient split conformal prediction region computing algorithm has been proposed under a general regression setting (Lei *et al.* 2018). We adapt the split conformal prediction algorithm to COC by first splitting the dataset $\left\{(x_{i},y_{i}),i=1,\ldots ,n\right\}$ into a training set $\left\{(x_{i},y_{i}),i\in I_{1}\right\}$ and a testing set $\left\{(x_{i},y_{i}),i\in I_{2}\right\}$. Without loss of generality, we assume that *n* is even such that $|I_{1}|=|I_{2}|=n/2$. We use the training set to fit a COC regression model to obtain $\hat{B}$. Then, we use the testing set to calculate conformity scores $R_{i}=\mathrm{KLD}(y_{i},\hat{B}x_{i})$, where $i\in I_{2}$. Let cutoff value *c* denote the ⌈(n/2+1)(1−α)⌉th smallest value in $\left\{R_{i}:i\in I_{2}\right\}$. For a new observation $x_{n+1}$, let $\hat{y}=\hat{B}x_{n+1}$, and the COC regression-based split conformal prediction region for this new observation $x_{n+1}$ is given by:

(6) $$C_{1-\alpha }^{\mathrm{split}}=\{y_{\mathrm{cand}}\in S^{q}:\mathrm{KLD}(y_{\mathrm{cand}},\hat{y})\leq c\}.$$

## 3 Results

We first designed simulations to evaluate performance of COC across a wide range of scenarios, including scenarios where model assumptions are violated. We further demonstrate its potential usefulness using two case study examples. One is to study relationships between paired microbiome compositions and the other is to investigate associations between bacterial and fungal residents in the gut microbiota.

### 3.1 Simulation: evaluation of point estimation and prediction

We first used two different distributions to generate compositional predictors. First, following Fiksel *et al.* (2022), we used Dirichlet distribution to simulate predictors $x_{i}∼$ Dirichlet$\left(1_{p}\right)$. Second, we simulated predictors following the simulation design of Lin *et al.* (2014) by first generating a latent n×p matrix $W=(w_{ij})$ from a multivariate normal distribution $N_{p}(\theta ,\Sigma )$, where $\theta ={\left(\theta_{1},\ldots ,\theta_{p}\right)}^{T}$ with $\theta_{j}=\text{log}(0.5p)$ for j=1,…,5 and $\theta_{j}=0$ otherwise, and $\Sigma =(\rho^{\left|i-j\right|})$ with ρ=0.5. Then, we obtained the compositional covariate matrix $X=(x_{ij})$ by $x_{ij}=\text{exp}(w_{ij})/\sum_{k=1}^{p}\;\text{exp}\;(w_{ik})$. The first scenario represents homogeneous compositional predictors where individual components are comparable to each other. The second scenario represents heterogeneous compositional predictors where some components (i.e. j=1,…,5) dominate the others.

We considered three different types of regression coefficient matrices in our simulation. In the first scenario, we randomly picked 1% of the columns in the **B**-matrix to be nonzero and set the remaining columns of **B** to $\frac{1}{q}1_{q}$. We used the Dirichlet ($1_{q}$) distribution to generate the nonzero columns, and we refer to this scenario as extremely sparse setting. In the second scenario, which we refer to as the sparse setting, 5% of the columns of **B** were simulated from Dirichlet ($1_{q}$) and the remaining columns were set to $\frac{1}{q}1_{q}$. In the third scenario, which we refer to as the dense setting, all columns of **B** were simulated from the Dirichlet ($1_{q}$) distribution. The sparsity assumption implied in the COC objective function (3) is violated under the dense scenario. Once both compositional predictors $x_{i}$ and regression coefficients matrix **B** were generated, we calculated the conditional expectation $E(y_{i}|x_{i})=Bx_{i}$ and then followed the strategy in the DR model (Fiksel *et al.* 2022) to simulate $y_{i}∼$ Dirichlet $\left(10\cdot E(y_{i}|x_{i})\right)$. After the data $(y_{i},x_{i}),i=1,\ldots ,n$ were simulated, we applied the proposed COC method and the DR (which has been referred to as DR hereafter for ease of presentation) method (Fiksel *et al.* 2022) to estimate the regression coefficient matrix $\hat{B}$. As far as we were concerned, other methods (Chen *et al.* 2017, Alenazi 2021) dealing with both compositional responses and compositional predictors rely on some sort of log-ratio transformations, and cannot directly handle excessive zeros in simulated data without a technical processing procedure (e.g. replacing zeros by a tiny positive value). Hence, under current simulation settings, it is more meaningful to only compare COC to DR, both of which are transformation-free.

To compare COC to DR, estimation and prediction accuracy were evaluated as follows. The estimation accuracy can be assessed by calculating the Frobenius norm $||\hat{B}-B|{|}_{F}$. To compare the prediction performance of two methods, we used the leave-one-out cross-validation strategy. Assuming sample *i* was preserved for prediction, we first used the remaining samples to obtain $\hat{B}$, and then found predicted responses ${\hat{y}}_{i}^{*}=\hat{B}x_{i}^{*}$. Next, the KLD between $y_{i}^{*}$ and ${\hat{y}}_{i}^{*}$ was calculated and then averaged over i=1,…,n. Throughout this simulation, we considered three sample sizes n=50,100 and set (p,q)=(500,10),(50,10), and (100,100) to mimic different sizes of compositional datasets. The point estimation and prediction results are reported in Tables 1 and 2, respectively.

**Table 1.** Average Frobenius norm error of estimation.

(*p*, *q*)	Predictor	Signal	*n*	COC	DR
			50	0.754 (0.033)	10.124 (0.115)
		E	100	0.641 (0.007)	13.824 (0.080)
			50	1.518 (0.025)	10.087 (0.123)
	HO	S	100	1.441 (0.006)	13.967 (0.079)
			50	6.374 (0.008)	13.273 (0.082)
		D	100	6.402 (0.007)	15.984 (0.055)
(500,10)					
			50	0.640 (0.007)	20.719 (0.006)
		E	100	0.637 (0.006)	19.955 (0.008)
			50	1.403 (0.006)	20.778 (0.007)
	HE	S	100	1.432 (0.008)	19.989 (0.010)
			50	6.365 (0.007)	21.629 (0.011)
		D	100	6.383 (0.008)	20.847 (0.012)
			50	0.272 (0.008)	3.396 (0.017)
		E	100	0.281 (0.008)	2.331 (0.012)
			50	0.395 (0.008)	3.380 (0.019)
	HO	S	100	0.395 (0.008)	2.310 (0.013)
			50	1.997 (0.007)	3.408 (0.020)
		D	100	2.002 (0.007)	2.237 (0.016)
(50,10)					
			50	0.276 (0.006)	5.258 (0.011)
		E	100	0.287 (0.007)	4.949 (0.010)
			50	0.396 (0.007)	5.255 (0.010)
	HE	S	100	0.387 (0.007)	4.974 (0.011)
			50	2.000 (0.008)	5.467 (0.013)
		D	100	2.020 (0.007)	5.171 (0.014)
			50	0.800 (0.004)	5.459 (0.024)
		E	100	0.557 (0.002)	3.847 (0.015)
			50	0.818 (0.003)	5.424 (0.018)
	HO	S	100	0.584 (0.002)	3.837 (0.015)
			50	1.169 (0.002)	5.539 (0.019)
		D	100	1.028 (0.001)	3.919 (0.002)
(100,100)					
			50	0.294 (0.003)	7.349 (0.009)
		E	100	0.108 (0.002)	6.947 (0.002)
			50	0.350 (0.003)	7.352 (0.009)
	HE	S	100	0.225 (0.001)	6.966 (0.012)
			50	1.020 (0.001)	7.398 (0.001)
		D	100	0.991 (0.001)	7.038 (0.011)

Mean values are calculated based on 100 replicates (standard errors of means are reported in parentheses). HO and HE stand for homogeneous and heterogeneous predictors, respectively. E, S, D stand for extremely sparse, sparse, and dense signals, respectively.

**Table 2.** Average leave-one-out (LOO) KLD between observed and predicted responses.

(*p*, *q*)	Predictor	Signal	*n*	COC	DR
			50	0.591 (0.021)	1.374 (0.116)
		E	100	0.537 (0.016)	1.908 (0.134)
			50	0.513 (0.029)	1.423 (0.109)
	HO	S	100	0.549 (0.018)	1.808 (0.112)
			50	0.736 (0.026)	1.817 (0.126)
		D	100	0.778 (0.028)	2.738 (0.179)
(500,10)					
			50	0.516 (0.021)	0.569 (0.023)
		E	100	0.525 (0.022)	0.564 (0.023)
			50	0.564 (0.023)	0.615 (0.027)
	HE	S	100	0.565 (0.022)	0.590 (0.023)
			50	0.481 (0.020)	0.515 (0.023)
		D	100	0.564 (0.022)	0.603 (0.024)
			50	0.682 (0.035)	1.538 (0.112)
		E	100	0.601 (0.023)	0.939 (0.056)
			50	0.642 (0.031)	1.652 (0.108)
	HO	S	100	0.569 (0.022)	0.944 (0.068)
			50	0.726 (0.033)	1.488 (0.109)
		D	100	0.710 (0.029)	1.037 (0.093)
(50,10)					
			50	0.534 (0.022)	0.604 (0.028)
		E	100	0.563 (0.021)	0.636 (0.027)
			50	0.530 (0.020)	0.615 (0.024)
	HE	S	100	0.535 (0.019)	0.585 (0.024)
			50	0.575 (0.023)	0.636 (0.027)
		D	100	0.588 (0.025)	0.605 (0.029)
			50	3.302 (0.093)	8.887 (0.352)
		E	100	3.123 (0.071)	6.752 (0.204)
			50	2.931 (0.054)	9.482 (0.372)
	HO	S	100	2.739 (0.041)	6.562 (0.242)
			50	2.784 (0.038)	9.046 (0.349)
		D	100	2.722 (0.040)	6.466 (0.274)
(100,100)					
			50	2.669 (0.032)	3.026 (0.047)
		E	100	2.662 (0.035)	2.914 (0.043)
			50	2.668 (0.036)	3.098 (0.057)
	HE	S	100	2.634 (0.027)	2.908 (0.038)
			50	2.692 (0.031)	3.017 (0.046)
		D	100	2.634 (0.031)	2.801 (0.044)

Mean values of *n* LOO KLDs are calculated (standard errors of means are reported in parentheses). HO and HE stand for homogeneous and heterogeneous predictors, respectively. E, S, D stand for extremely sparse, sparse, and dense signals, respectively.

As shown in Table 1, estimation performance of COC is consistently better than the DR method across all considered simulation scenarios. For example, when (p,q)=(500,10) with heterogeneous compositional predictors, the estimation error of COC is around 0.64 and that of DR is around 20.5, which is 30 times larger than that of COC. The proposed COC estimator has a smaller Frobenius norm than DR for both homogeneous and heterogeneous predictors, as well as for the extremely sparse and sparse scenarios. This is true even in the dense scenario, where the underlying sparsity assumption is violated. However, the average Frobenius norms are smaller when the association signals are sparser, which indicates that COC does have better performance in presence of sparse signals.

The pattern for prediction analysis displayed in Table 2 is slightly different. With homogeneous compositional predictors, COC predictions are significantly better than those of DR, which is consistent with we have observed in Table 1. For example, when (p,q)=(500,10) with heterogeneous compositional predictors, the prediction error of COC is around 0.55 and that of DR is around 2.40. However, with heterogeneous compositional predictors, the discrepancy between COC and DR is no longer substantial based on numerical results shown in Table 2. In our data generation scheme, a heterogeneous compositional vector is dominated by its first five components while values of the remaining (p−5) components are close to zero. Note that the predicted value of a new observation $x^{*}$ is ${\hat{y}}^{*}=\hat{B}x^{*}$. Thus, when transferred to predicted values, the gained estimation accuracy of COC over DR on many columns of **B** (as indicated in numerical results shown in Table 1) is reduced because most components of $x^{*}$ are very close to zero. Therefore, the gain in prediction performance of COC regression over DR is not as impressive as that in estimation with heterogeneous compositional predictors.

Besides point estimation and prediction, we have conducted additional simulation studies to demonstrate the superior and robust performance of COC. In particular, we also designed numerical experiments to compare COC to log-ratio transformation-based methods under a different data generating setting, where the key COC model assumption E(y|x)=Bx is violated. Numerical results of these additional simulations are presented in Section B of the online supplementary data at *Bioinformatics* online.

### 3.2 Evaluation of inference

Simulation studies were also used to study the effectiveness of the permutation tests proposed by Fiksel *et al.* (2022) in comparison to the proposed bootstrap hypothesis test given in Algorithm 1. We assess the empirical Type-I error rates and power of two methods for determining if all the elements of **B** are equal to 1/q, i.e. if $H_{0}:B=B_{0}$. The permutation test of DR was implemented by using the function codalm_indep_test() from the R codalm package (Fiksel *et al.* 2022), and this approach uses the DR model to estimate the **B** matrix. We considered a high-dimensional case where p=q=100. The predictors were generated under the homogeneous and heterogeneous cases. To evaluate Type-I error rates, we set each column of **B** to be $\frac{1}{q}1_{q}$. In this case, the signal is labeled as 0 in Table 3. We also evaluated the power in the sparse (S) and dense (D) cases, which are identical scenarios to those used to produce Tables 1 and 2. In all cases, the data were simulated 100 times, and the empirical power (or Type-I error rate in the case of no signal) was estimated as the proportion of times of rejecting $H_{0}$.

**Table 3.** Empirical Type-I error rates and powers for testing $H_{0}:B=B_{0}$ for p=q=100 using Algorithm 1 (COC) and the permutation test from Fiksel *et al.* (2022) (DR).

Predictor	Signal	*n*	COC	DR
		50	0.01	0.06
	0 (Type-I error)	100	0.02	0.05
		50	0.44	0.06
HO	S	100	0.80	0.11
		50	0.57	0.54
	D	100	1.00	1.00
		50	0.00	0.03
	0 (Type-I error)	100	0.00	0.02
		50	1.00	0.00
HE	S	100	1.00	0.05
		50	1.00	0.02
	D	100	1.00	0.03

A significance level of α=0.05 was used. S and D stand for sparse and dense signals, respectively.

Overall, Table 3 shows that the COC test (described in Algorithm 1) has more power than the permutation test (labeled as DR) from Fiksel *et al.* (2022) in the sparse and dense cases. In these cases, $B\neq B_{0}$ and $H_{0}$ should be rejected. The Type-I error rates for both tests tend to be at most α, with the exception of the permutation test for n=50, which had a Type-I error rate slightly higher than α=0.05. Notably, the permutation test has very low power in the simulated cases when the predictors are heterogeneous, while the proposed bootstrap test always rejects $H_{0}$ in these cases. The improvement of test performance of COC over DR is probably because COC has much more accurate model estimates than DR in high-dimensional settings.

We next conducted simulation studies to assess the proposed COC regression-based conformal prediction inference procedure described in the main text. We followed previous settings to generate compositional predictors **x** under both homogeneous and heterogeneous cases. We considered the most general case of a dense coefficient matrix **B** and used this to generate the compositional responses. We fixed the sample size at n=100 and set the nominal level 1−α=0.95 for the prediction region given in Equation (6). To evaluate coverage rates of the conformal prediction region, a total of *p* points were selected near the center of the simplex $S^{p}$. The goal of this simulation was to see how often the conformal prediction regions captured the true response for each of these *p* points. Each new observation ($x_{n+1}$) was obtained by taking the center point ${\left(1/p,\ldots ,1/p\right)}^{T}$ of $S^{p}$ and adding δ to the $j^{th}$ coordinate for j=1,…,p, where δ=0.01,0.1. These points were then renormalized so that they sum to 1. Each of these points was added to a simulated dataset of size n=100 from either the homogeneous case or the heterogeneous case, and then the resulting 101 responses (including the new $y_{n+1}$) were simulated accordingly. The data were randomly split into training and testing sets of equal size. We then recorded if the true response $y_{n+1}$ was in the 95%-prediction region. This procedure was repeated 50 times for each point, and the coverage rate for each point was calculated as the proportion of times that true response $y_{n+1}$ being in the conformal prediction region calculated from Equation (6). Finally, we calculated the average coverage rate for all points and reported them in Table 4.

**Table 4.** Average coverage for predicted compositional responses $y_{n+1}$ from new compositional predictors $x_{n+1}$ near center of *p*-dimensional simplex for a 95% conformal prediction region.

(*p*, *q*)	δ	Homogeneous	Heterogeneous
(500,10)	0.01	0.9988	0.9732
	0.1	0.9952	0.9764
(50,10)	0.01	0.9976	0.9672
	0.1	0.9980	0.9652
(100,100)	0.01	0.9792	0.9884
	0.1	0.9720	0.9872

Higher values of δ indicate that predictors are further away from the center.

As shown in Table 4, the prediction regions maintain at least an average coverage of 95% across all scenarios being considered. Under most scenarios, the coverage rate is higher when δ=0.01, where smaller values of δ imply that the points used to evaluate coverage are closer to the center of the simplex. When p>q, the coverage is higher in the homogeneous case, likely because all of the components of the predictor composition are treated similarly under the Dirichlet model. Overall, the proposed conformal prediction regions have good coverage rates on the true compositional responses.

### 3.3 Application: analysis of paired microbiome compositions

In microbiome research, it is common to collect paired data to study the underlying biological mechanism. One such example is the mucosal microbiome data collected from inflammatory bowel disease patients (Morgan *et al.* 2015). A total of 255 samples, including 196 from prepouch ileum (PPI) tissues and 59 from pouch, were collected and 51 subjects had paired PPI and pouch samples. Since two tissue locations (PPI and pouch) are close to each other, it is of interest to question whether there is correlation between mucosal microbiome compositions of one tissue and the other. A COC analysis of regressing PPI compositions on pouch compositions can provide solutions to this type of question and shed insights into how microbial communities of different human body locations interacts with each other.

**Figure 1.**
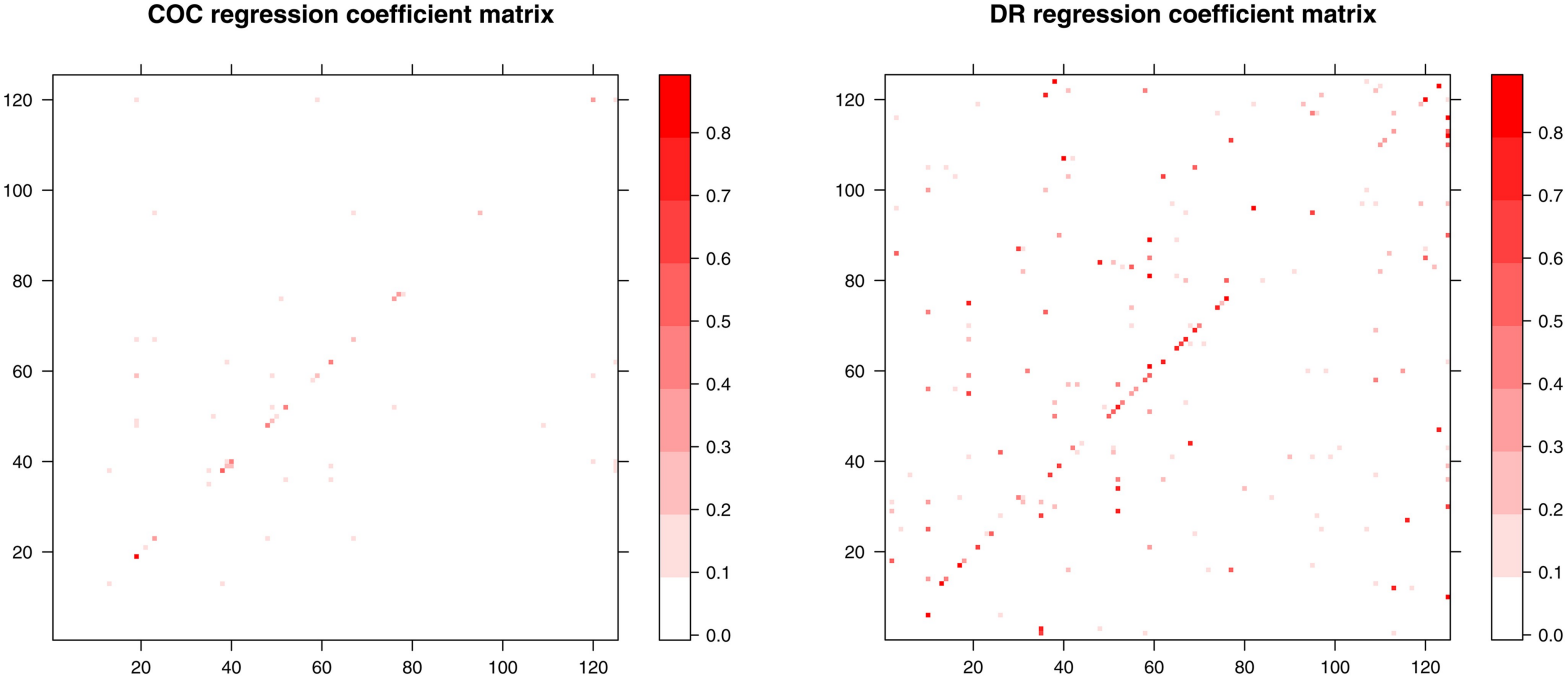
Heatmap of estimated coefficient matrix $\tilde{B}$ by COC and DR. The *X* and *Y* axes represent indices of components in the compositional vector.

The original mucosal microbiome data contain 7000 OTUs, with many singletons that were only detected in a single sample. To both mitigate spurious effects of potential sequencing errors or sample contamination and reduce computational burden, we first amalgamate these 7000 OTUs into genus level by aggregating relative abundances of all species within a same genus and then adopted the same criterion used in the original analysis (Morgan *et al.* 2015) to select taxa with more than three counts in more than three samples in subsequent statistical analysis, which left us a compositional vector with 125 genera. Using the 51 paired samples of these genera compositions, both COC and the DR method of Fiksel *et al.* (2022) were applied to analyze the paired PPI-pouch microbiome compositions. Due to the Markov matrix constraint on **B**, it is more meaningful to visualize $\tilde{B}=B-1/q$, whose heatmaps are displayed in Fig. 1.

As shown in Fig. 1, COC lead to a much more parsimonious regression model compared to DR, which is expected based on the COC objective (Equation (3)). The nonzero regression coefficient of COC mostly appear on the diagonal which implies that PPI microbiome compositions and pouch microbiome compositions are quite concordant. This is consistent with the conclusion drawn in the original investigation (Morgan *et al.* 2015) using totally different analysis tools. On the other hand, the estimated regression coefficient matrix of DR is dense and a bit irregular, which makes it difficult for pattern interpretation. For the estimated COC regression coefficient matrix $\hat{B}$, 53 out of the 125 columns are all $\frac{1}{125}$’s, which indicates a sparsity level above 40% in the sense of sparsity used in our simulation studies. We calculated pairwise differences $||B_{.j}-B_{.j^{\prime }}|{|}_{2}^{2}$ and found out that columns corresponding to the Escherichia genus and unclassified genus have the largest difference, which implies the joint effect of these two taxa has the largest impact on responses. Besides interpretation and visualization of regression coefficients, we also compared prediction performance of COC and DR on this paired PPI-pouch data. We adopted the leave-one-out strategy to reserve one sample as the testing set and the remaining 50 paired samples as the training set to estimate **B**. Then, using the pouch compositions of the reserved testing point, we predicted its PPI compositions. The KLD between predicted pouch compositions and observed pouch compositions can be calculated. We rotate this procedure for each of the 51 pairs to obtain 51 KLDs for both COC method and DR method. The average KLDs of COC and DR are 0.604 and 0.658, respectively. The Wilcoxon signed rank test *P*-value of the differences is .0027, which indicates that our COC method does have a significantly better prediction performance than DR on this PPI-pouch data under the nominal level of 0.05. In summary, the proposed COC method stands out in obtaining a parsimonious model with better model interpretation and prediction performance than the existing DR method.

### 3.4 Application: fungi bacteria association analysis

In this analysis, we aim to study the relationship between bacterial and fungal residents in the gut microbiota based on gut bacterial and fungi composition data generated from the COMBO study (Wu *et al.* 2011). The COMBO study is a cross-sectional study of the dietary effect on the human gut microbiota based on 98 subjects. Stool samples were collected and DNA samples were analyzed by the 454/Roche pyrosequencing of 16S rDNA gene segments of the V1–V2 region for the bacterial composition and ITS1 rDNA gene segment for fungal composition. The pyrosequences were denoised prior to read clustering. The denoised sequences were then quality-controlled and analyzed by the QIIME pipeline (Caporaso *et al.* 2010). OTUs were formed at 97% and 95% similarity for bacterial and fungal sequences, respectively, with the default parameter settings. Taxonomy was assigned to OTU representative sequences using the RDP classifier (Cole *et al.* 2009) for bacterial sequences and BROCC (Dollive *et al.* 2012) for fungal sequences. A total of 3068 and 290 nonsingleton OTUs were detected for the bacterial and fungi part of the gut microbiota, respectively. To reduce the sparsity level, we further aggregated OTU counts to the genus level for bacteria and the class level for fungi based on the OTU taxonomy. We included p=40 bacterial genera (treated as compositional predictors) and q=7 fungi classes (treated as compositional responses) that appeared in at least 10% subjects, and removed samples with less than 100 fungal reads. After intersection of the two datasets, 90 samples were included in the analysis. Our goal is to identify the bacterial genera that are associated with the fungal classes based on the two compositional datasets.

**Figure 2.**
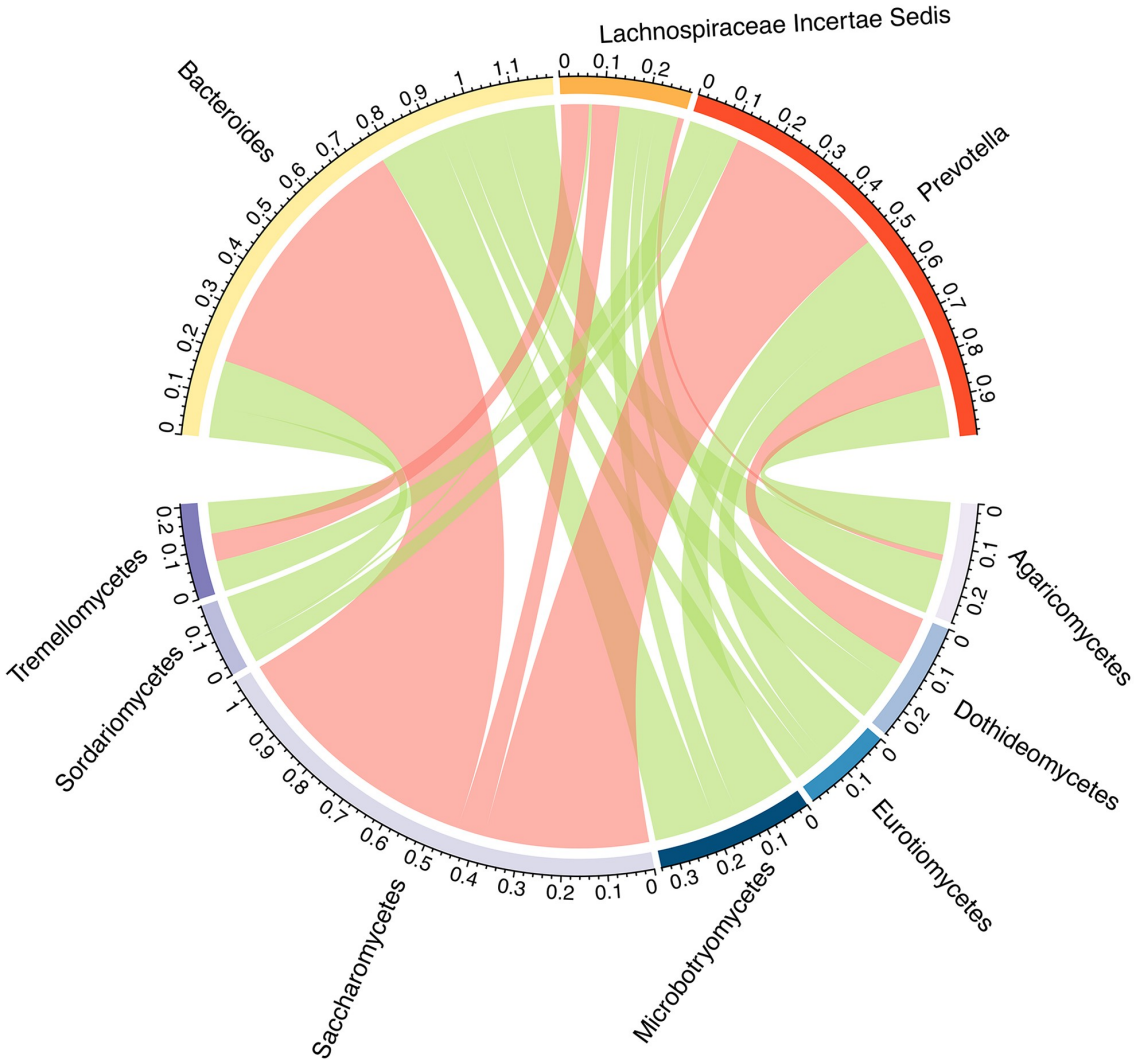
The relationships between bacterial genera and fungal classes learned from COC. The thickness of connecting lines indicates the magnitude of coefficients and pink and green color indicate positive and negative association, respectively.

Applying COC to regress fungi compositions on bacteria compositions, we identified three bacterial genera (*Prevotella, Bacteriodes, Lachnospiraceae I.S.* as shown in the top part of Fig. 2) that were associated with all seven fungal classes (bottom part of Fig. 2). The *Prevotella/Bacteriodes* ratio was found to be important for the gut microbiome structure and was also shown to be significantly associated with the overall fungi compositions (Hoffmann *et al.* 2013). Our results recapitulated the same observation that *Prevotella* and *Bacteriodes* play a pivotal role in shaping the fungal community structure as they are densely associated with all seven fungal classes. As shown in Fig. 2, the Saccharomycetes class has the strongest positive associations with both *Prevotella* and *Bacteriodes* among the seven fungal classes. Saccharomycetes, where the *Candida* is the dominant genus, is able to degrade starches, especially after pretreatment with amylases (Hoffmann *et al.* 2013) such as the human-encoded amylases present in the mouth and small intestine. Thus, Saccharomycetes may assist in breaking down starch in carbohydrate rich foods, which in turn liberates simpler sugars to be fermented by bacteria such as *Prevotella*. Taken together, our re-analysis of the microbial composition data from the COMBO study reveals a clear and clean interactions between the bacterial and fungal residents in the gut microbial community.

**Figure 3.**
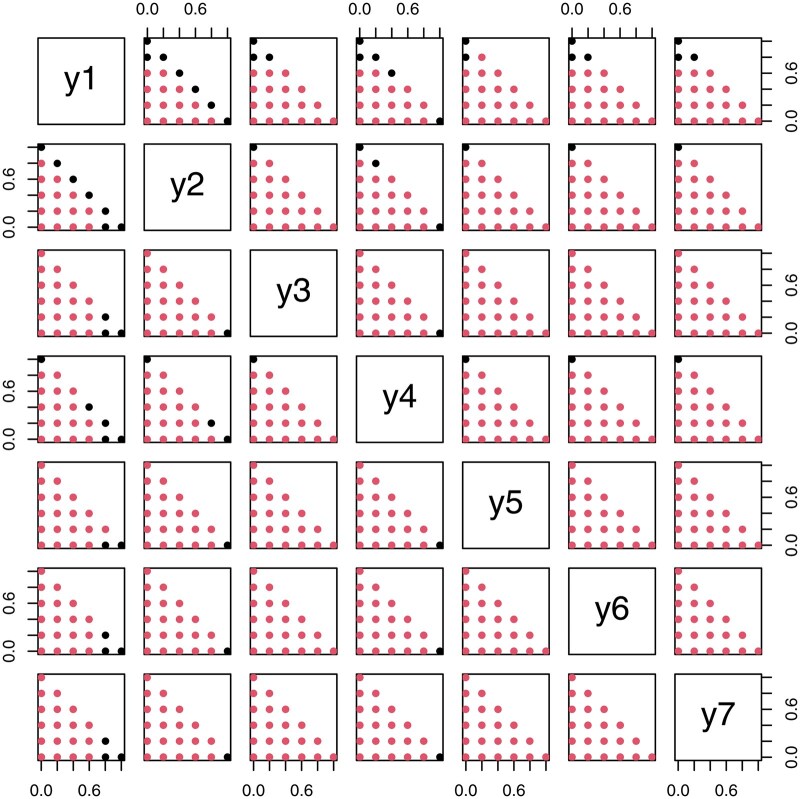
Illustration of 95% prediction region coverage for fungi composition at a new bacterial composition in the testing set. Red points are in the prediction region and black points are not.

We next randomly split 90 samples into a training set and a testing set to illustrate the split conformal prediction analysis. For ease of presentation, let $y_{1},\ldots ,y_{7}$ denote Agaricomycetes, Dothideomycetes, Eurotiomycetes, Microbotryomycetes, Saccharomycetes, Sordariomycetes, Tremellomycetes, respectively. Figure 3 shows the coverage information of the 95% prediction region for the fungi compositions ${\left(y_{1},y_{2},\ldots ,y_{7}\right)}^{T}$ given a new observation of a bacterial compositional vector. The prediction region is visualized through all $\left(\begin{array}{c} 7\\ 2 \end{array}\right)$ possible projections of the response simplex $S^{7}$ onto two dimensions. In each panel, black points are not included in the prediction region. For example, Fig. 3 shows that given this specific predictor, large proportion of $y_{1}$, extremely large proportion of $y_{2}$ and $y_{4}$ are excluded from the prediction region. Additionally, fungal compositions that are mostly composed of $y_{1},y_{2}$ are excluded from the prediction region, as indicated by the black upper diagonal in the subpanel corresponding to coordinates $y_{1}$ and $y_{2}$. This suggests that, given these specific bacterial compositions, the concurrent abundance of Agaricomycetes ($y_{1}$) and Dothideomycetes ($y_{2}$) is improbable. Therefore, COC offers valuable insights into co-occurrence patterns between bacterial and fungal inhabitants in the gut ecosystem.

## 4 Discussion

Compositional data are frequently encountered in many scientific disciplines nowadays and regression analysis of such data would enable us to examine relationships between two or more compositions of interest, which can further lead to scientific insights. Surprisingly, statistical methods for regression analysis with both responses and predictors being compositional are less developed, especially in the high-dimensional settings. Building upon the transformation-free DR framework (Fiksel *et al.* 2022), we have introduced a novel penalized estimation equation approach that is both biologically meaningful and statistically powerful in that it enables more accurate model estimation and prediction in a high dimensional setting, such as compositional data collected from a NGS experiment as considered in this article. Compared to DR, the performance gain of COC in estimation and prediction in high-dimensional regression analysis is substantial especially when compositional predictors are homogeneous and responses-predictors association signals are sparse as demonstrated in our numerical studies. We employ COC to microbiome compositional data analysis to illustrate its potential usefulness and superior performance in this article and envision COC has great potential for application to other types of sequence count data, which might be compositional if the total abundances are lost and only relative abundance information has been carried during the sequence counting measurement process (Vandeputte *et al.* 2017).

Compared to traditional log-ratio-based analysis, COC does not have subcompositional coherence (Aitchison 1982). On the other hand, since zero measurements are widely observed in high-throughput NGS-based biological and biomedical research, the fact that COC can accommodate zeros in both responses and predictors gives it a huge advantage in applications to these zero-inflated datasets over traditional log-ratio transformation-based methods. While subcompositional coherence has long been viewed as a desired property in the compositional data analysis community, there is recent evidence indicating that this may not be a serious concern in high-dimensional settings in that the subcompositional incoherence becomes diluted when the number of parts is large (Greenacre 2021). Besides the proposed transformation-free COC method, the recent Box-Cox type chiPower transformation (Greenacre 2024) also provides a versatile alternative to log-ratio-based methods to cope with zeros. It is of interest to investigate properties of the chiPower transformation in compositional data analysis in future research. Currently, the COC model assumes a linear relationship between responses and predictors, which can be limited in presence of nonlinear association patterns. Also, the penalty function we have introduced in COC equally weights each component of the predictors. A weighted penalty scheme could be developed if additional information on the importance of predictors are available. Some of the extensions to address these potential limitations of COC are relatively straightforward, while others may warrant further investigation.

## Supplementary Material

btaf387_Supplementary_Data

## Data Availability

Data analyzed in this paper are publicly available from previous publications (Morgan *et al.* 2015, Wu *et al.* 2011).
