# Supplementary material for: Composition-on-composition regression analysis for multi-omics integration of metagenomic data
Source: Bioinformatics. 2025 Jul 12;41(7):btaf387. doi: 10.1093/bioinformatics/btaf387 (PMC12279295; doi:10.1093/bioinformatics/btaf387)
Supplement: btaf387_Supplementary_Data [file btaf387_supplementary_data.pdf]

Supplementary material for Composition-On-Composition regression analysis for multi-omics integration of metagenomic data

Abstract

This supporting information file consists of two parts: (A) A computing algorithm to fit the COC regression model; (B) Additional simulation results under model misspecification.

A A computing algorithm for COC estimation

To solve the following optimization problem in COC regression:

$$\hat{\mathbf{B}} = \underset{\mathbf{B}\in\mathcal{F}}{\operatorname{argmin}} - \sum_{i=1}^{n}\sum_{k=1}^{q} y_{ik} \log\left(\sum_{j=1}^{p} x_{ij}B_{kj}\right) + \lambda \sum_{j=1}^{p}\sqrt{\sum_{k=1}^{q}\tilde{B}_{kj}^2}, \tag{1}$$

we use the augmented Lagrangian method to handle constraints in $\mathbf{B}$ (Bertsekas, 2014). Problem (1) can be converted to an equivalent optimization problem by minimizing the corresponding augmented Lagrangian function as follows:

$$\begin{aligned} L(\mathbf{B},\boldsymbol{\gamma}) = &-\frac{1}{n}\sum_{i=1}^{n}\sum_{k=1}^{q} y_{ik}\log\left(\sum_{j=1}^{p} x_{ij}B_{kj}\right) + \lambda\sum_{j=1}^{p}\sqrt{\sum_{k=1}^{q}(B_{kj}-\frac{1}{q})^2} \\ &+\frac{\theta}{2}\sum_{j=1}^{p}\left(\sum_{k=1}^{q}B_{kj}-1\right)^2 + \sum_{j=1}^{p}\gamma_j\left(\sum_{k=1}^{q}B_{kj}-1\right), \end{aligned} \tag{2}$$

where θ is a fixed parameter and $\boldsymbol{\gamma} = (\gamma_1, \ldots, \gamma_p)$ are Lagrange multipliers. At iteration s with parameter values $\mathbf{B}^{(s)}, \gamma_j^{(s)}, j = 1, \ldots, p$, we obtain the estimates of $\mathbf{B}, \boldsymbol{\gamma}$ sequentially at iteration $(s+1)$ as follows:

$$\mathbf{B}^{(s+1)} = \underset{\mathbf{B}}{\operatorname{argmin}}\, L(\mathbf{B}, \boldsymbol{\gamma}^{(s)}), \tag{3}$$

$$\gamma_j^{(s+1)} = \gamma_j^{(s)} + \theta\left(\sum_{k=1}^{q} B_{kj}^{(s+1)} - 1\right),\ j = 1, \ldots, p. \tag{4}$$

Subproblem (3) can be solved by the proximal gradient descent method, and the jth column of $\mathbf{B}$ is updated by

$$\mathbf{B}_{.j}^{(s+1)} = \left[(1 - \frac{\lambda t^{(s)}}{||\mathbf{B}_{.j}^{(s)} - t^{(s)}g(\mathbf{B}_{.j}^{(s)}) - q^{-1}\mathbf{1_q}||})_+(\mathbf{B}_{.j}^{(s)} - t^{(s)}g(\mathbf{B}_{.j}^{(s)}) - \frac{1}{q}\mathbf{1_q}) + \frac{1}{q}\mathbf{1_q}\right]_+, \tag{5}$$

where $(u)_+ = \max(u, 0)$, $\mathbf{1_q}$ is a q-dimensional column vector with all elements being 1, $t^{(s)}$ is the algorithm step-size and the kth component of $g(\mathbf{B}_{.j}^{(s)})$ is

$$g_k(\mathbf{B}_{.j}^{(s)}) = -\frac{1}{n}\sum_{i=1}^{n}\frac{x_{ij}y_{ik}}{\sum\limits_{j=1}^{p} x_{ij}B_{kj}^{(s)}} + \theta\sum_{k=1}^{q}(B_{kj}^{(s)} - 1) + \gamma_j^{(s)}.$$

We now summarize our computing algorithm to fit COC regression in Algorithm 1.

Algorithm 1 Proximal gradient descent method of multipliers

1. Initialize $\tilde{\mathbf{B}}^{(0)}$ with 0 or a warm start, $\theta > 0$, $s = 0$ and $\gamma_j^{(0)} = 0$, $j = 1, \ldots, p$.
2. For $j = 1, \ldots, p$, $k = 1, \ldots, q$, update $B_{kj}^{(s+1)}$ by (5) until convergence.
3. For $j = 1, \ldots, p$, update $\gamma_j^{(s+1)}$ by (4).
4. Update $s \leftarrow s + 1$, and repeat Steps 2 and 3 until convergence. Output $\hat{\mathbf{B}} = \mathbf{B}^{(s+1)}$.

Remark: To ensure compliance with the inequality constraints $B_{kj} \geq 0$, which are intrinsic to the feasible region $\mathcal{F}$, we employ the operator $(\cdot)_+$ as described in equation (5). As a result, the minimization process of problem (3) executed in Step 2 of Algorithm 1 may not achieve exact minimization, and the feasible region $\mathcal{F}$ might only contain an approximate minimizer for problem (3). Previous studies have confirmed the convergence of Algorithm 1 under conditions of inexact minimization (Lin et al., 2014). Readers who are interested in this topic can refer to Proposition 1 on Page 5 of Lin et al. (2014).

Another thing about the computing algorithm is selection of tuning parameter. The objective function (2) has two tuning parameters λ and θ, which need to be selected when implementing Algorithm 1 for COC regression analysis. The tuning parameter λ controls the trade-off between Kullback–Leibler divergence (KLD) and complexity of the composition-on-composition regression. Here we use K-fold cross validation with $K = 5$ or 10 to select the optimal λ parameter. In particular, we split the whole data set $(\mathbf{X}, \mathbf{Y}) := \{(\mathbf{x}_i, \mathbf{y}_i)\}_{i=1}^{n}$ into K non-overlapping subsets of nearly equal size $(\mathbf{X}^{(k)}, \mathbf{Y}^{(k)}), k = 1, \ldots, K$. Each time we preserve the kth part and use the remaining data to fit the COC regression model to first obtain $\hat{B}^{(-k)}$ and then evaluate the KLD between $\mathbf{Y}^{(k)}$ and $\mathbf{X}^{(k)}\hat{B}^{(-k)}$. Using K-fold cross validation, We select the optimal λ by solving

$$\hat{\lambda} = \underset{\lambda \geq 0}{\operatorname{argmin}} \sum_{k=1}^{K}\sum_{i=1}^{n_k} KLD(\mathbf{y}_i^{(k)}, \mathbf{x}_i^{(k)}\hat{B}^{(-k)}),$$

where n_k is the sample size of $(\mathbf{X}^{(k)}, \mathbf{Y}^{(k)})$ and $(\mathbf{x}_i^{(k)}, \mathbf{y}_i^{(k)})$ denotes the ith sample in $(\mathbf{X}^{(k)}, \mathbf{Y}^{(k)})$. The tuning parameter θ is needed to enforce equation constraints in $\mathbf{B}$ (i.e., sum column sums to one) and does not affect convergence of Algorithm 1 as long as $\theta > 0$. In practice, we take $\theta = 1$ throughout our numerical studies and find out that estimated columns $\hat{\mathbf{B}}_{.j}$'s are reasonably close to a compositional vector within an affordable computing time.

We have developed our COC model estimation computing algorithm in absence of additional covariates besides compositional responses and predictors. In practice, it's also conceivable that sometimes additional covariates or potential confounders, such as common biological or clinical

variables like age and gender, may influence both compositional responses and predictors. Therefore, adjusting for these covariates' effects is crucial when examining the relationship between compositional responses and predictors. Let $\mathbf{C}$ represent the design matrix for these covariates, incorporating the intercept term as its first column. We posit that the quantity of such covariates is relatively small to ensure that $\mathbf{P}_C = \mathbf{C}(\mathbf{C}^T\mathbf{C})^{-1}\mathbf{C}^T$ forms the well-defined projection matrix into the column space of $\mathbf{C}$. Denoting the nth order identity matrix by $\mathbf{I}$, where n signifies the sample size, $\mathbf{Y}$ as the $n \times q$ response matrix, and $\mathbf{X}$ as the $n \times p$ predictor matrix, we define $\tilde{\mathbf{Y}} = (\mathbf{I} - \mathbf{P}_C)\mathbf{Y}$ and $\tilde{\mathbf{X}} = (\mathbf{I} - \mathbf{P}_C)\mathbf{X}$ as the covariates adjusted response and predictor matrices, respectively. Post-adjustment, the rows of $\tilde{\mathbf{X}}$ and $\tilde{\mathbf{Y}}$ cease to be compositional vectors, yet it can be verified that each row sums to zero. As long as the sums of each row of $\tilde{\mathbf{X}}$ and each row of $\tilde{\mathbf{Y}}$ are identical, we still need each column of the regression coefficient matrix sums to one to make sure that the corresponding regression model is well-defined. That is, while a similar condition as equation (2) of the main text remains necessary for the regression coefficients between $\tilde{\mathbf{Y}}$ and $\tilde{\mathbf{X}}$, the requirement for non-negativity can be relaxed. Thus, we can lift the restrictions of non-negative coefficients in matrix $\mathbf{B}$ by making corresponding changes in the COC computing algorithm to perform the covariates adjusted COC regression analysis between $\tilde{\mathbf{Y}}$ and $\tilde{\mathbf{X}}$.

B Additional simulations: evaluation of prediction performance under model misspecification

We also evaluated our method under a model misspecification scenario. This was done to see if the proposed COC method performs well when the data were generated by a very different model. In particular, we did not use the conditional mean structure $E(\mathbf{Y}|\mathbf{X}) = \mathbf{BX}$ to generate response variables. The true compositional responses were generated using an Additive Log-Ratio (ALR) model. This is a more traditional approach to modeling compositional data (Aitchison, 1982). This model has the form

$$E[\text{alr}(\mathbf{y})_k \mid \text{alr}(\mathbf{x})] = \beta_{0k} + \sum_{j=1}^{p-1} \beta_{jk}\text{alr}(\mathbf{x})_j, \quad k = 1, \dots, (q-1), \tag{6}$$

where $\text{alr}(\mathbf{x})_j = \log(x_j/x_p)$ for $j = 1, \dots, (p-1)$, and $\text{alr}(\mathbf{y})_k = \log(y_k/y_q)$ for $k = 1, \dots, (q-1)$. In Model (6), $\boldsymbol{\beta}_k := (\beta_{0k}, \dots, \beta_{(p-1)k}) \in \mathbb{R}^p$ for each $k = 1, \dots, (q-1)$, so this model does not assume that the coefficients are compositional, which makes it quite different from the COC regression model presented in the main text. We picked the ALR model as an illustration of model misspecification in this simulation. The ALR model was chosen because a similar ALR model was previously used in the simulation studies of the DR method (Fiksel et al., 2022). Other log-ratio transformations-based model are also possible candidates of misspecified models.

Since zero values were not allowed in the log-ratio transformation, we slightly modified our data generation scheme described previously in Section 3.1 of the main text. As before, the compositional covariates $\mathbf{X}$ were generated under the same homogeneous and heterogeneous scenarios described in Section 3.1 of the main text. To avoid taking the natural logarithm of zero, a very small positive value (1×10^{-30}) was added to all values of the design matrix $\mathbf{X}$ before the additive log-ratio transformation. As done previously, three levels of sparsity in the model coefficients were considered. In the first scenario, 1% of the coefficients were randomly generated from a $N(1, 0.5)$ distribution, and the remaining coefficients were set to 0. The second scenario assumed only 5% of the coefficients were nonzero, and the last scenario assumed all coefficients were nonzero using the $N(1, 0.5)$ distribution. Next, mean-zero normal errors with variance 0.01 were added to $\beta_{0k} + \sum_{j=1}^{p-1} \beta_{jk}\text{alr}(\mathbf{x})_j$ to

obtain the simulated additive log-ratios of responses $alr(\mathbf{Y})$. Finally, the compositional responses $\mathbf{Y}$ were calculated from $alr(\mathbf{Y})$ by applying the inverse log-ratio transformation:

$$y_k = \frac{\exp(alr(y_k))}{\sum_{k=1}^{q-1} \exp(alr(y_k)) + 1}, k = 1, \ldots, (q-1), \;\; y_q = \frac{1}{\sum_{k=1}^{q-1} \exp(alr(y_k)) + 1}.$$

After the data were simulated, the COC, DR and ALR regression models were fit to the data. The ALR regression analysis directly takes the additive log-rations as predictors and responses in a multivariate linear regression model and is one of the earliest approaches used in the literature to conduct regression analysis with both compositional responses and compositional predictors (Fiksel et al., 2022). Since the sample size n is much smaller than the number of predictors p, naively fitting the ALR model (6) to the simulated training data via least squares will result in poor predictive performance. To deal with this high-dimensional issue, a LASSO penalty was used for regularization when fitting the ALR model to the training dataset in these simulations. This is equivalent to applying the composition Lasso method proposed in Lin et al. (2014) multiple times to each $alr(\mathbf{y})_k, k = 1, \ldots, (q-1)$. Since representations of regression coefficients in COC or DR and ALR regression (as in model (6)) are different, comparisons of regression coefficient estimation performance is meaningless. Hence, we focus on comparisons of model predictions. Same as Table 2 of the main text, the average KLD between $\hat{\mathbf{y}}_i^*$ and $\mathbf{y}_i^*$ (i.e., on the testing set) was used to compare the performance of COC, DR, and the ALR model in terms of prediction accuracy.

Table S1 shows the average KLD between the observed and predicted responses for the COC, DR, and ALR models over 100 replicates, when the responses were generated by the ALR model (6). Standard errors of those mean values reported in Table S1 were similar to those reported in the Table 1 and 2 of the main text and are hene omitted for ease of presentation. Similar to what we have observed in Table 2 of the main text, when the compositional predictors are homogeneous, the proposed COC method has better predictive accuracy (i.e. lower mean KLD) than DR. In the homogeneous cases, DR performs worse than ALR, while COC has similar performance to ALR in these cases. It is expected that the ALR will perform reasonably well, since the true model is an ALR model. When the predictors are heterogeneous, both COC and DR have similar performance. The average KLD for ALR greatly increases when $q = 100$, as opposed to the scenarios where $q = 10$. When $q = 100$, COC has lower mean KLD than ALR. When $q = 100$ and the compositional predictors are heterogeneous, DR also has lower mean KLD than ALR. Overall, even when the model is misspecified, the proposed COC method has a robust prediction performance and tends to be the best method or close to the best one among the three methods being evaluated across a wide range of scenarios.

References

Aitchison, J. (1982). The statistical analysis of compositional data. *Journal of the Royal Statistical Society: Series B (Methodological) 44*(2), 139–160.

Bertsekas, D. P. (2014). *Constrained optimization and Lagrange multiplier methods.* Academic press.

Fiksel, J., S. Zeger, and A. Datta (2022). A transformation-free linear regression for compositional outcomes and predictors. *Biometrics 78*(3), 974–987.

Lin, W., P. Shi, R. Feng, and H. Li (2014). Variable selection in regression with compositional covariates. *Biometrika 101*(4), 785–797.

Table S1: Average KLD between observed and predicted responses when true response follows an ALR model. Mean values are calculated based on 100 replicates. HO and HE stand for homogeneous and heterogeneous predictors, respectively. E, S, D stand for extremely sparse, sparse and dense signals, respectively.

(p, q)	Predictors	Signals	n	COC	DR	ALR
			50	1.099	2.126	1.973
		E	100	1.157	2.607	1.127
			50	1.866	4.759	2.138
	HO	S	100	1.799	4.808	1.777
			50	0.453	1.244	0.434
		D	100	0.422	1.353	0.410
(500,10)						
			50	1.325	1.325	1.558
		E	100	1.313	1.316	1.546
			50	2.085	2.104	2.572
	HE	S	100	2.062	2.059	2.567
			50	1.264	1.278	1.818
		D	100	1.236	1.234	1.843
			50	2.113	4.144	2.205
		E	100	2.077	3.171	2.202
			50	1.769	2.829	2.027
	HO	S	100	1.653	2.269	1.991
			50	1.738	2.628	2.029
		D	100	1.583	2.000	2.032
(50,10)						
			50	1.183	1.138	1.549
		E	100	1.145	1.054	1.559
			50	1.917	1.851	2.596
	HE	S	100	1.932	1.836	2.462
			50	1.984	1.940	2.569
		D	100	1.925	1.877	2.357
			50	3.548	7.045	3.647
		E	100	3.241	5.845	3.432
			50	2.601	3.677	3.183
	HO	S	100	2.474	3.448	3.373
			50	2.470	2.768	3.436
		D	100	2.107	2.668	3.377
(100,100)						
			50	1.765	1.763	2.613
		E	100	1.727	1.689	2.646
			50	3.207	3.288	5.137
	HE	S	100	3.136	3.166	5.159
			50	2.757	2.746	4.176
		D	100	2.524	2.511	3.997
